# Characterizations of Polymer Gears Fabricated by Differential Pressure Vacuum Casting and Fused Deposition Modeling

**DOI:** 10.3390/polym13234126

**Published:** 2021-11-26

**Authors:** Chil-Chyuan Kuo, Ding-Yang Li, Zhe-Chi Lin, Zhong-Fu Kang

**Affiliations:** 1 Department of Mechanical Engineering, Ming Chi University of Technology, No. 84, Gungjuan Road, New Taipei City 243, Taiwan; M10118002@mail.mcut.edu.tw (D.-Y.L.); U09117212@mail.mcut.edu.tw (Z.-C.L.); U09117222@mail.mcut.edu.tw (Z.-F.K.); 2 Research Center for Intelligent Medical Devices, Ming Chi University of Technology, No. 84, Gungjuan Road, New Taipei City 243, Taiwan

**Keywords:** polymer gear, additive manufacturing, differential pressure vacuum casting, polyurethane resin, abrasion

## Abstract

In recent years, polymer gears have gradually become more widely employed in medium or heavy-duty conditions based on weight reduction in transmission systems because of low costs and low noise compared to metal gears. In the current industry, proposing a cost-effective approach to the manufacture of polymer gears is an important research issue. This paper investigates the wear performance of polymer gears fabricated with eight different kinds of materials using differential pressure vacuum casting and additive manufacturing techniques. It was found that both additive manufacturing and differential pressure vacuum casting seem to be an effective and cost-effective method for low-volume production of polymer gears for industrial applications. The gate number of one is the optimal design to manufacture a silicone rubber mold for differential pressure vacuum casting since the weld line of the polymer is only one. Polyurethane resin, 10 wt.% glass fiber-reinforced polylatic acid (PLA), or 10 wt.% carbon fiber-reinforced PLA are suggested for manufacturing gears for small quantity demand based on the deformation and abrasion weight percentage under process conditions of 3000 rpm for 120 min; epoxy resin is not suitable for making gears because part of the teeth will be broken during abrasion testing.

## 1. Introduction

In practice, product developers need to overcome a tricky issue by making a small batch of prototypes for testing economy and feasibility. A gear is a rotating circular machine part, which can change the torque, speed, and direction of a power source in industrial applications. The polymer gear has some distinct advantages compared to the metal gear, including low weight, quietness of operation, and no need for external lubrication [1], and has been widely used in the automotive industry and consumer electronics. Additive manufacturing (AM) [2,3] has been defined as the process of building physical models by joining materials layer upon layer using computer numerical control data. The application of AM processes has increased in fabricating physical models across various industries because of its capability in manufacturing functional parts with complex geometries. Thus, the AM technology has been widely used to produce prototypes or physical models since it has the capacity to manufacture components with sophisticated geometric shapes. Ghelloudj et al. [4] developed an engineering model to express the evolution of tooth flank wear in polyamide spur gears as a function of the number of cycles. It was found that a wear correction parameter was added to compensate for the measuring errors when plotting the wear profile curves. The simulation results are in good agreement with those obtained from experimental measurements. Lu et al. [5] detected the injection molding lunker defects by X-ray computed tomography. Results showed that the lunker defect jeopardizes the loading capacity of the tooth root under medium or heavy loading conditions, while the tooth flank failure is significantly influenced by the loading condition. Zhang et al. [6] optimized the performance of 3D-printed gears using a machine learning process using a genetic algorithm-based artificial neural network multi-parameter regression model; the authors found that the wear performance of 3D-printed gears was increased by three times. Vacuum casting (VC) [7,8] is a promising technique used for the production of functional parts due to its fast production of high-quality prototypes. Oleksy et al. [9] manufactured the gear wheels with epoxy composites using VC technology and found that developed multi-stage homogenized hybrid-filled epoxy resin had a regular layered morphology. Furthermore, the tensile strength was increased by up to 44 %. Kai et al. [10] integrated VC and AM as well as rapid tooling for fabricating connectors. It was found that a stereolithography apparatus mold cannot be used directly in the VC process since the stereolithography apparatus mold must be broken into pieces for extracting the molded parts. Puerta et al. [11] proposed a new approach to determine the suitability of the usage of standard tensile test specimens fabricated by VC and fused deposition modeling (FDM). The results revealed that the surface quality of the model used for the creation of the silicone rubber mold is an important issue in the VC. Zhang et al. [12] proposed a differential pressure technology to improve the quality of resin parts using VC technology through the optimization method. The results revealed that the artificial fish-swarm algorithm optimized the response surface model of the warpage via the optimized process parameters. Zhao et al. [13] manufactured an accurate shark-skin surface in a large area to overcome some difficulties in the replication process via VC technology. It was found that process parameters played an important role in eliminating air bubbles on the surface of the resin parts. Frankiewicz et al. [14] demonstrated the results of analyses performed for the process of replicating mechanoscopic marks with the use of three vacuum-casting variants, including a hybrid vacuum-pressure casting process developed in particular for the purposes of replication. It was found that the proposed method not only allowed the tool preparation to be simplified and shortened, but also caused the entire process time to be shortened from 10 to 1.5 h.

Injection molding and machine cutting are normally used to fabricate polymer gears. However, the use of plastic injection molding to manufacture polymer gears requires a set of steel injection molds, which does not seem to be a good approach during the research and development stage of a new polymer gear. A set of cutting tools is required for machining polymer gears by machine cutting. Note that these methods are suitable for mass production of polymer gears based on cost-effectiveness. Therefore, developing a cost-effective method for batch production of polymer gears in the research and development stage is an important research issue. In general, the integration of silicone rubber mold and vacuum casting technology [15] is widely used for rapid manufacturing prototypes since the silicone rubber mold has elastic and flexible characteristics. Accordingly, a prototype with complex geometries can be fabricated easily [16]. Chu et al. [17] proposed an efficient generation grinding method for a spur face gear along the contact trace using a disk CBN wheel. Results demonstrated that the proposed method breaks new ground for the engineering application of face gears.

Vacuum casting is a cost-effective method used for the low-volume production of physical models. However, conventional vacuum casting employs the gravity of molding material to fill the mold cavity, resulting in some common defects, such as insufficient filling, shrink marks, or trapped air observed in the cast. Especially, these defects can be eliminated using differential pressure vacuum casting (DPVC) [18]. Therefore, the end-use prototypes can fundamentally be formed by silicone rubber mold using DPVC. The advantages of manufacturing polymer gears using AM techniques include design freedom and less waste of materials. However, not much work has been conducted to characterize the differences in polymer gears fabricated by AM and DPVC. The goal of this investigation is to investigate the characterizations of polymer gears fabricated by AM and DPVC techniques using eight different kinds of polymers. In addition, in-house abrasion testing equipment was designed and implemented to evaluate spur gear life. Finally, an effective and cost-effective method for the low-volume production of polymer gears was proposed.

## 2. Materials and Methods

**Figure 1:**
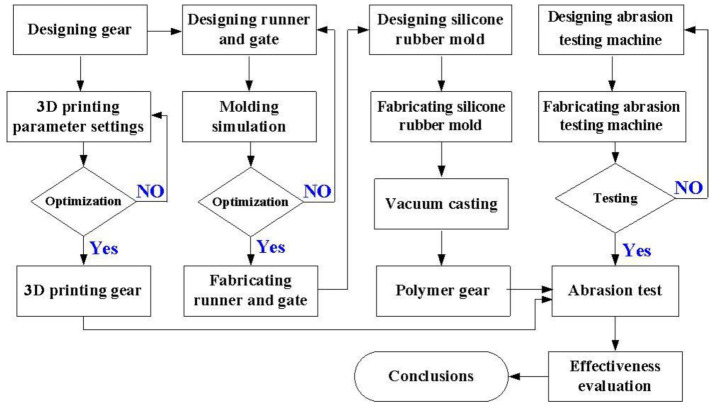
Research process of this study.

Figure 1 shows the research process of this study. The gear type selected in this study is a spur gear since this is the simplest type of gear. Firstly, two spur gears were designed using computer-aided design (CAD) software (Cero, parametric technology corporation Inc.. Taipei, Taiwan), i.e., driving gear and passive gear.

**Figure 2:**
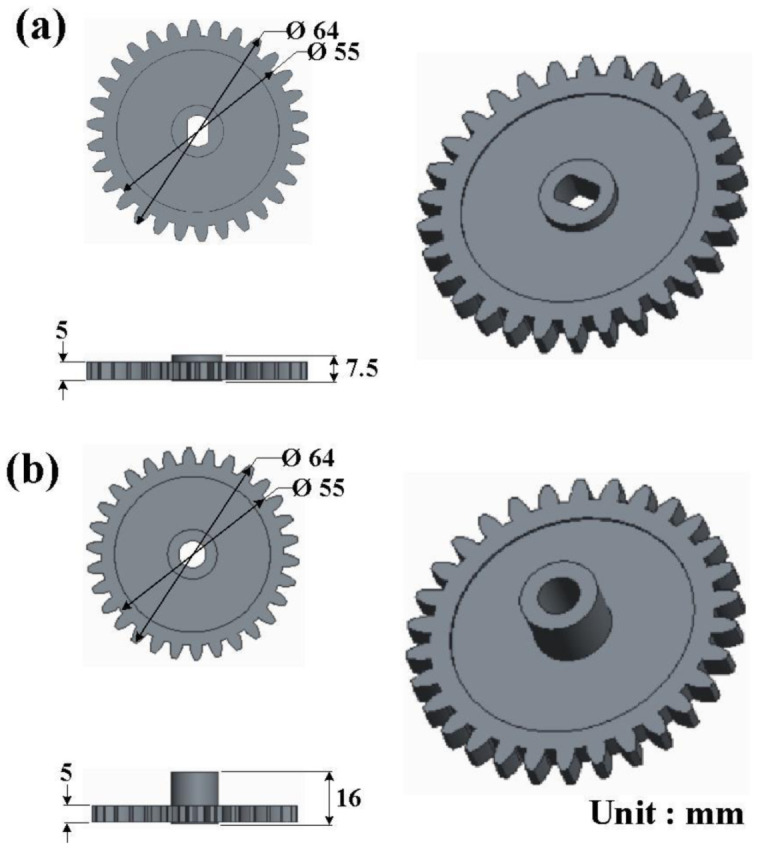
3D CAD model and dimensions of (**a**) driving gear and (**b**) passive gear.

Figure 2 shows a three-dimensional (3D) CAD model and the dimensions of the driving gear and the passive gear. The number of teeth, pitch diameter, tooth module, pressure angle, and thickness of the gear are 30, 60 mm, 2 mm, 20° and 5 mm, respectively. 

**Figure 3:**
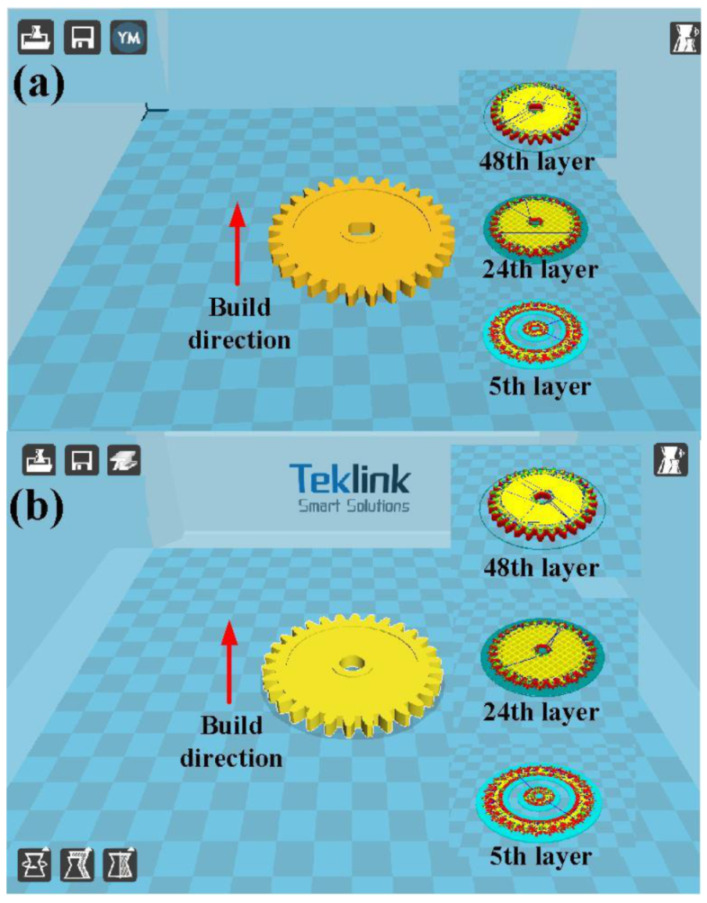
3D printing software interface of (**a**) driving gear and (**b**) passive gear.

Figure 3 shows the 3D printing software interface of the driving gear and the passive gear. Designing the runner system for the silicone rubber mold is crucial to the mold design. Conventionally, designing the runner system significantly depends on the mold designer’s experiences. To address these issues, the filling system of the silicone rubber mold is investigated using numerical simulation software. To investigate the optimum filling system of the silicone rubber mold, the 3D CAD models of spur gear, runner, and gate were imported to the Moldex3D simulation software (R16SP3OR, CoreTech System Inc., Hsinchu, Taiwan) via a data exchange STEP format. Table 1 shows the main numerical modeling parameters used in the numerical analysis.

**Figure 4:**
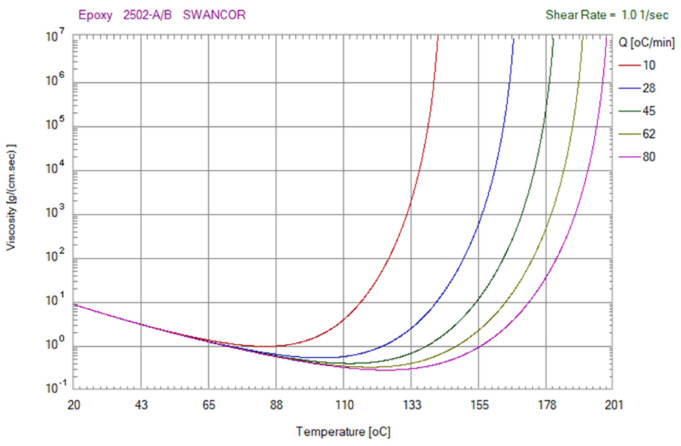
Viscosity as a function of the temperature of the epoxy molding material.

**Figure 5:**
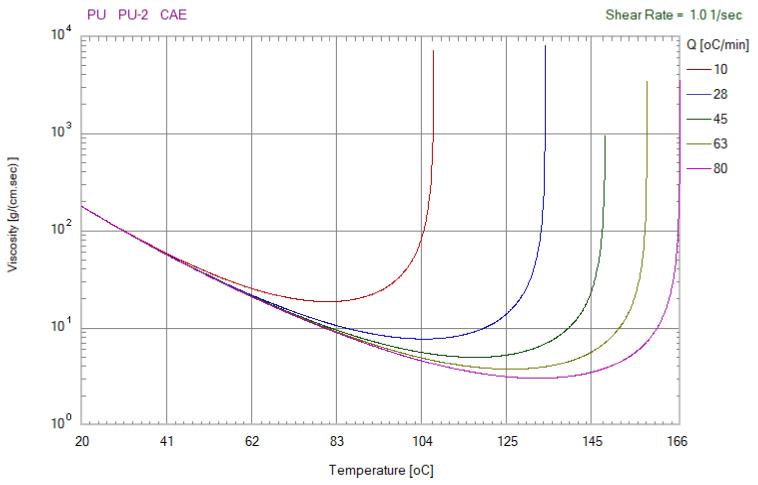
Viscosity as a function of the temperature of the polyurethane molding material.

**Figure 6:**
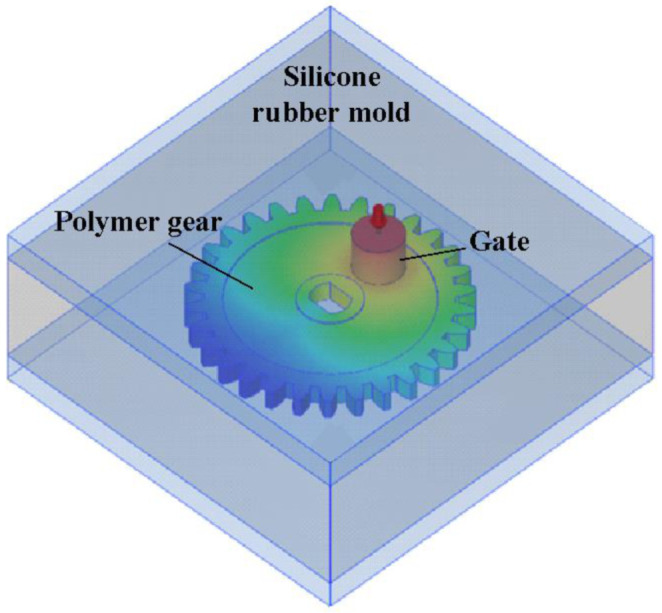
Relationship between filling system, cast part, and silicone rubber mold.

Figure 4 shows the viscosity as a function of the temperature of the epoxy molding material. Q stands for temperature ramping rate of the mixture. Figure 5 shows the viscosity as a function of the temperature of the polyurethane (PU) molding material. In this study, a standard sprue–runner–gate system was used due to the low pressure drop during DPVC. Thus, the pouring materials can flow directly into the silicone rubber mold cavity without passing through the intricate runner system. Figure 6 shows the relationship between the filling system, cast part, and the silicone rubber mold.

**Figure 7:**
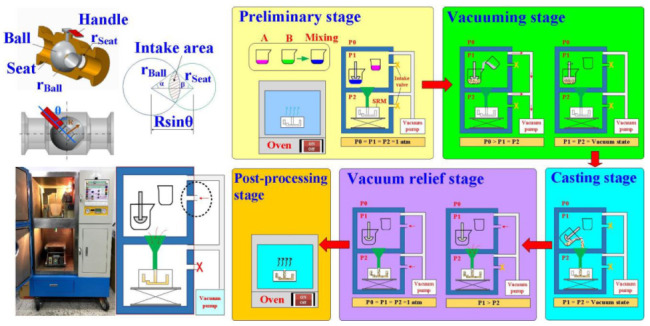
Five stages of the VC and information about ball value and intake area.

Figure 7 shows the five stages of the VC and information about ball value and intake area. In general, the VC process involves five distinct stages: preliminary, vacuuming, casting, vacuum relief, and post-processing stages. The P1, P2, and P3 stand for mixing chamber pressure, casting chamber pressure, and atmospheric pressure, respectively. The preliminary stage is the preparation of the silicone rubber mold based on the size of the gear prototype. The radii of ball valve, ball, and seat are 15 mm, 7.5 mm, and 6.25 mm, respectively. In this study, a room temperature vulcanization liquid silicone rubber (KE-1310ST, Shin Etsu Inc, Hsinchu, Taiwan) was used to fabricate the silicone rubber mold. The base compound and hardener (CAT-1310S, Shin Etsu Inc.) were mixed in a weight ratio of 10: 1. A vacuum casting machine (F-600, Feiling Inc., Taoyuan, Taiwan) was used to remove air bubbles in the mixture resulting from the mixing process under vacuum conditions. The epoxy and polyurethane resins were selected as casting materials to fabricate spur gears by silicone rubber mold using differential pressure vacuum casting technology. The process parameters for manufacturing gears include a ball valve angle of 60 °, a silicone rubber mold preheating temperature of 27 °C, a molding material mixing time of 30 s, a pouring time of 40 s, and a differential pressure time of 20 s. The spur gears were also manufactured using an FDM machine (Infinity X1E, Photonier Inc., Taipei, Taiwan) with a nozzle diameter of 0.4 mm. In this study, the six different kinds of filaments, i.e., virgin polylactic acid (PLA) (Thunder 3D Inc., Taipei, Taiwan), PLA filled with 10 wt.% glass fiber (Thunder 3D Inc.), PLA filled with 10 wt.% carbon fiber (Thunder 3D Inc.), acrylonitrile butadiene styrene (ABS) (Thunder 3D Inc.), polycarbonate (PC), and polyamide (PA) were used to print polymer gears using the FDM technique according to the standard of ASTM52900. The process parameters for printing spur gears with a PLA filament are printing temperature of 200 °C, hot bed temperature of 60 °C, printing speed of 50 mm/s, and layer thickness of 0.1 mm. The process parameters for printing spur gears with both PLA filled with 10 wt.% glass fiber and 10 wt.% carbon fiber filaments are printing temperature of 200 °C, hot bed temperature of 70 °C, printing speed of 50 mm/s, and layer thickness of 0.1 mm. The process parameters for printing spur gears with ABS, PC, and PA filaments are printing temperature of 100 °C, hot bed temperature of 60 °C, printing speed of 50 mm/s, and layer thickness of 0.1 mm. The infill density was set as 100%. The Ultimaker Cura software (New Taipei, Taiwan) was used to generate the program for the FDM machine. Chemical compositions of six different kinds of filaments were characterized using energy-dispersive x-ray spectroscopy (EDS) (D8 ADVANCE, Bruker Inc., Karlsruhe, Germany) and field-emission-scanning electron microscopy (FE-SEM) (JEC3000-FC, JEOL Inc., Tokyo, Japan).

**Figure 8:**
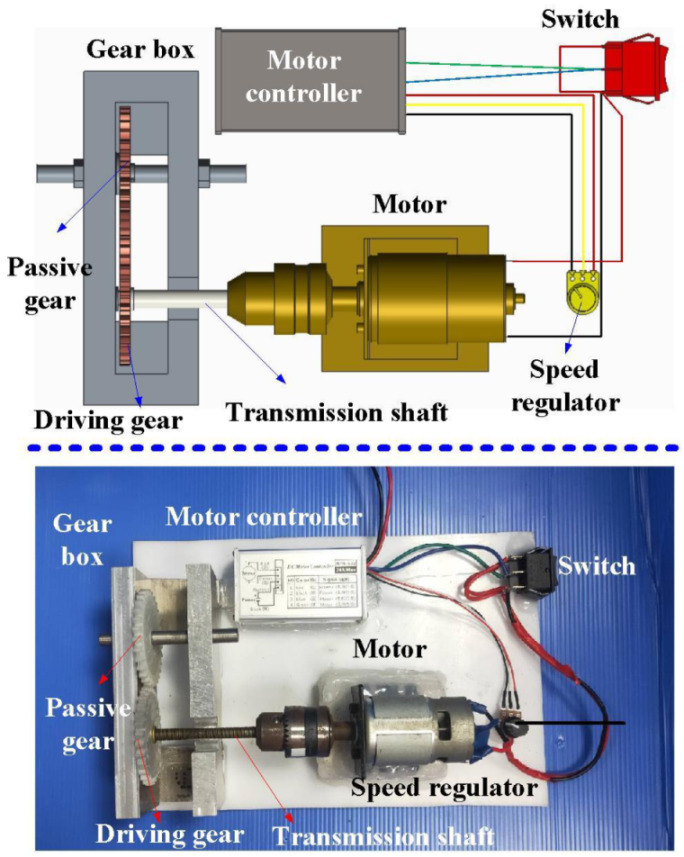
A gear abrasion testing machine developed in this study.

Tool wear is the main factor contributing to tool failure in cutting difficult-to-machine materials [19]. Similarly, the abrasion rate is the main factor causing spur gear failure. Various methods, including cylinder-on-plate [20], block-on-wheel, pin-on-disk [21], block-on-ring, pin-on-plate, or flat-on-flat can be used to investigate the wear rate. However, these methods require several testing conditions. In this study, a simple gear abrasion testing equipment was designed and implemented for investigating the wear performance of the fabricated polymer gears. Figure 8 shows a gear abrasion testing machine developed in this study. The tooth flank wear of spur gears as a function of the number of cycles was investigated. Corner wear evolution of gears fabricated with eight different materials was investigated using an OM (M835, Microtech, Inc., Dresden, Germany). The deformation angles of the printed spur gears were measured using a vision measuring system (Quick Vision 404, Mitutoyo Inc., Gunpo, Korea).

## 3. Results and Discussion

**Figure 9:**
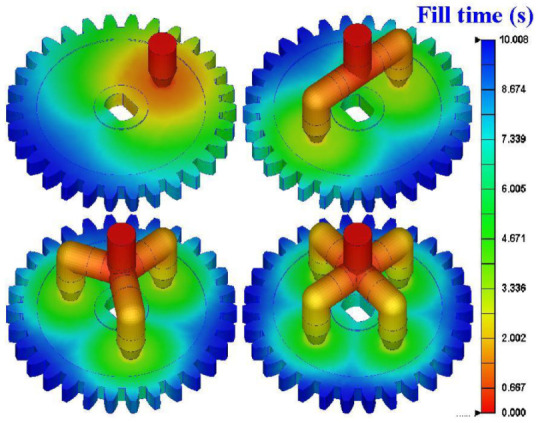
Filling results of different gate numbers.

The efficiency, yield, or product quality of the vacuum casting was affected by the design of the pouring gate. The most common defects such as air-traps or short shot will occur due to poor filling in the vacuum casting. The shrinkage or warpage of the cast part will occur due to unbalanced flow. The post-processing time and costs will increase due to incorrect gate size or location. To avoid these disadvantages described above, the Moldex3D molding simulation software was utilized to investigate the most suitable gate for vacuum casting. There are four different gate types: single point, two points, three points and four points. These gate types were investigated for the gear design in vacuum casting. Figure 9 shows the filling results of different gate numbers. It was found that the gears can be filled completely for four different gate numbers. The fill times for gate numbers of one, two, three, and four are all about 10 s.

**Figure 10:**
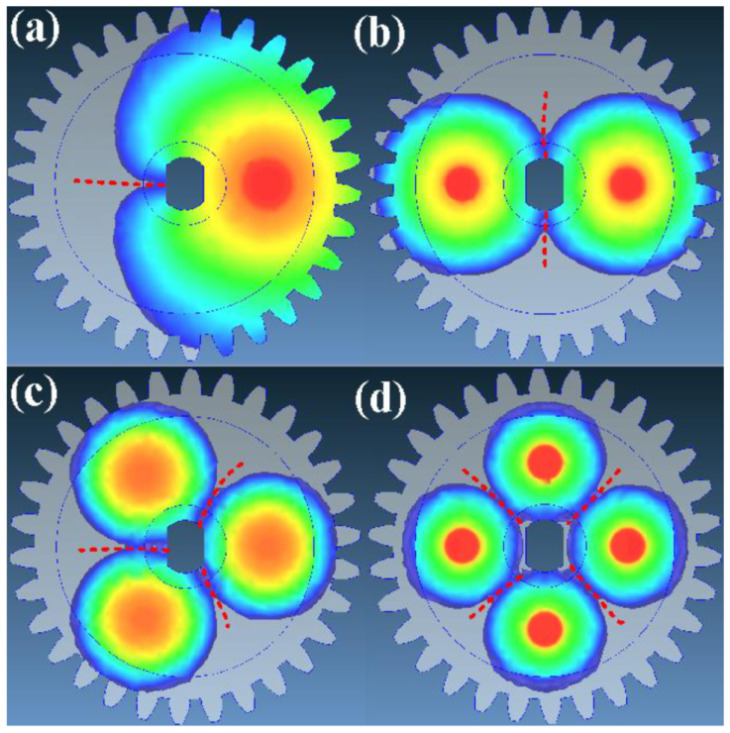
Weld line results for different gate numbers of (**a**) single point, (**b**) two points, (**c**) three points, and (**d**) four points.

**Figure 11:**
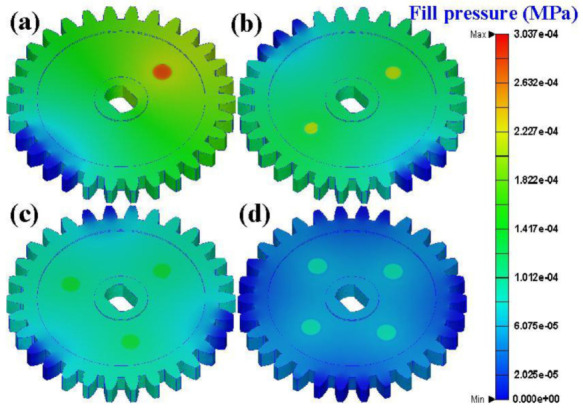
Filling maximum pressures for gate numbers of (**a**) single point, (**b**) two points, (**c**) three points, and (**d**) four points.

Figure 10 shows the weld line results for different gate numbers. The weld lines are formed by two different melt fronts joining together during the filling stage, which significantly reduces the strength of the molded part. Figure 11 shows the filling maximum pressures for different gate numbers. The filling maximum pressures for gate numbers of a single point, two points, three points, and four points are 1.439 × 10^−4^ MPa, 1.035 × 10^−4^ MPa, 8.441 × 10^−5^ MPa, and 4.272 × 10^−5^ MPa, respectively. The maximum filling pressure decreases with as the number of gates increases. It should be noted that the differences in filling pressure can be ignored since the material was poured in a vacuum environment.

**Figure 12:**
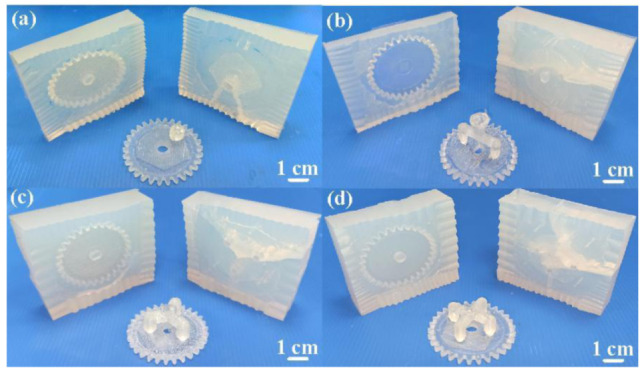
Silicone rubber molds for gate numbers of (**a**) single point, (**b**) two points, (**c**) three points, and (**d**) four points.

Figure 12 shows the silicone rubber molds with different gate numbers. As can be seen, the number of weld lines for gate numbers of one, two, three, and four are one, two, three, and four, respectively. Based on practical experience, fewer weld lines represent a better the quality of gears. In addition, the post-processing time and costs of the cast parts for the gate number of one were less than those of the cast parts made with gate numbers of two, three, and four. According to the results described above, the single-point gate seems to be the optimal gate number to fabricate a silicone rubber mold for DPVC.

**Figure 13:**
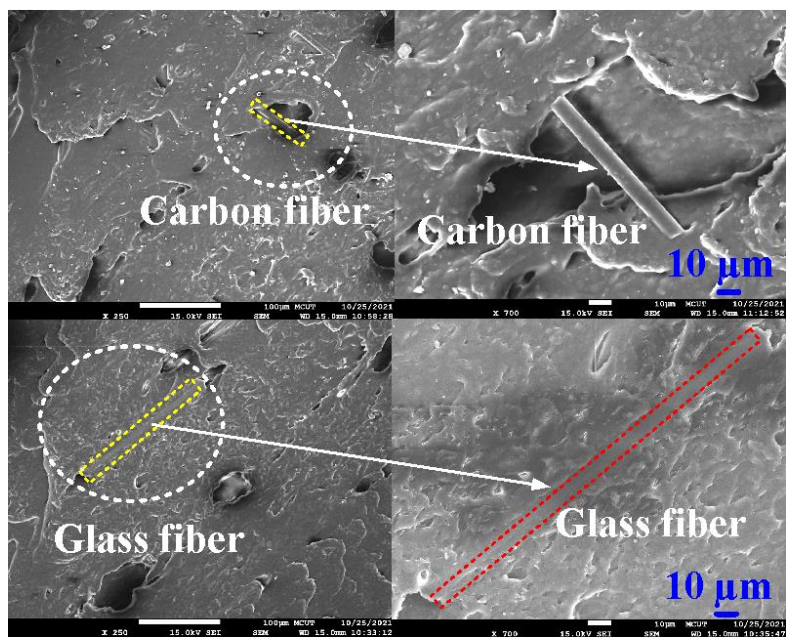
FE-SEM images of 10 wt.% glass fiber-reinforced PLA and 10 wt.% carbon fiber-reinforced PLA.

**Figure 14:**
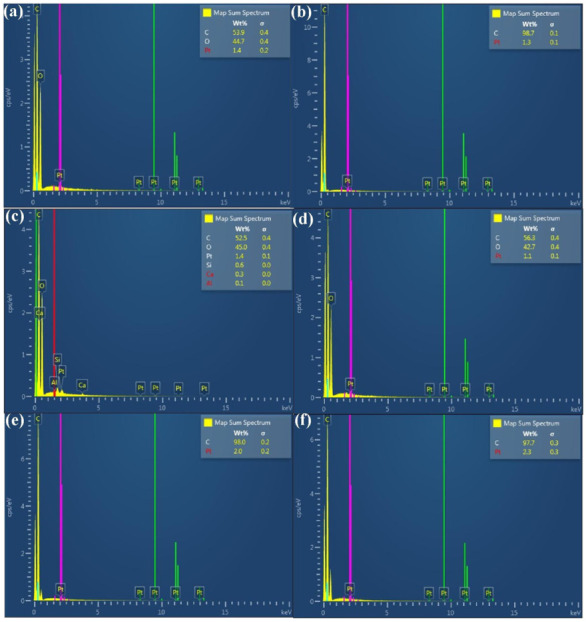
EDS analysis of (**a**) PLA, (**b**) ABS, (**c**) 10 wt.% glass fiber-reinforced PLA, (**d**) 10 wt.% carbon fiber-reinforced PLA, (**e**) PA, and (**f**) PC filaments.

**Figure 15:**
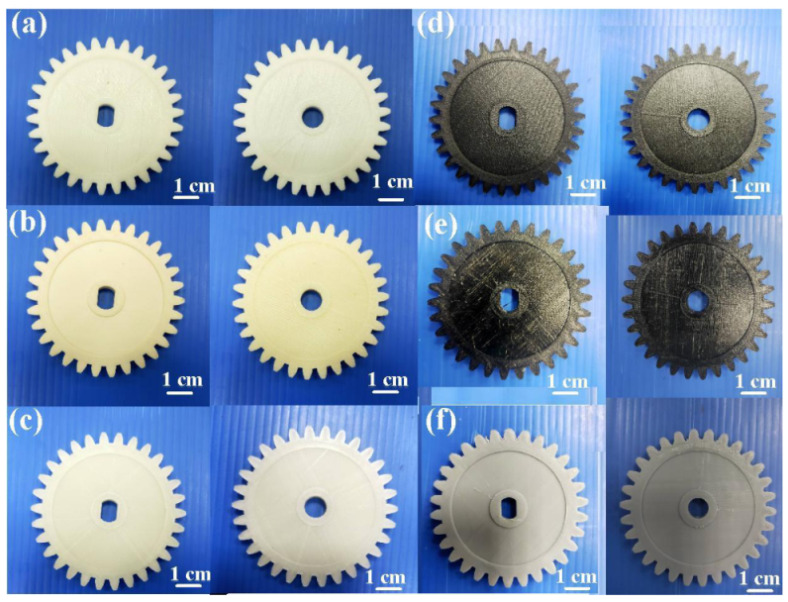
Typical spur gears fabricated with six different filaments of (**a**) PLA, (**b**) ABS, (**c**) 10 wt.% glass fiber-reinforced PLA, (**d**) 10 wt.% carbon fiber-reinforced PLA, (**e**) PA, and (**f**) PC using FDM technique.

Figure 13 shows FE-SEM images of 10 wt.% glass fiber-reinforced PLA and 10 wt.% carbon fiber-reinforced PLA. This result indicates that glass fiber or carbon fiber was observed in the filaments applied to fabricate polymer gears using the FDM technique. Impurity was not observed, which was also confirmed by EDS element mapping analysis. Figure 14 shows EDS analysis of PLA, ABS, 10 wt.% glass fiber-reinforced PLA, 10 wt.% carbon fiber-reinforced PLA, PA, and PC filaments. The major compositions of PLA, ABS, 10 wt.% carbon fiber-reinforced PLA, PA, and PC filaments are C and O. In particular, components of 10 wt.% glass fiber-reinforced PLA are Si, C, O, Ca, and Al. Figure 15 shows the spur gears fabricated with filaments of PLA, ABS, 10 wt.% glass fiber-reinforced PLA, 10 wt.% carbon fiber-reinforced PLA, PA, and PC using the FDM technique.

**Figure 16:**
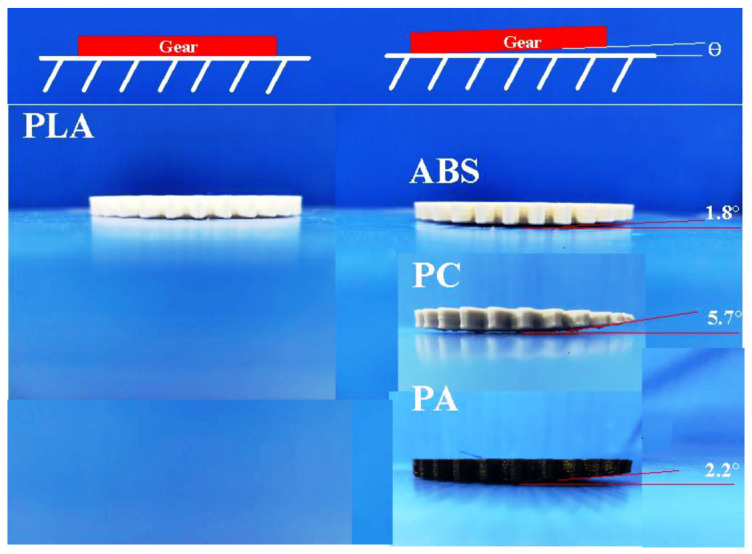
Spur gears printed with PLA, ABS, PC, and PA filaments.

**Figure 17:**
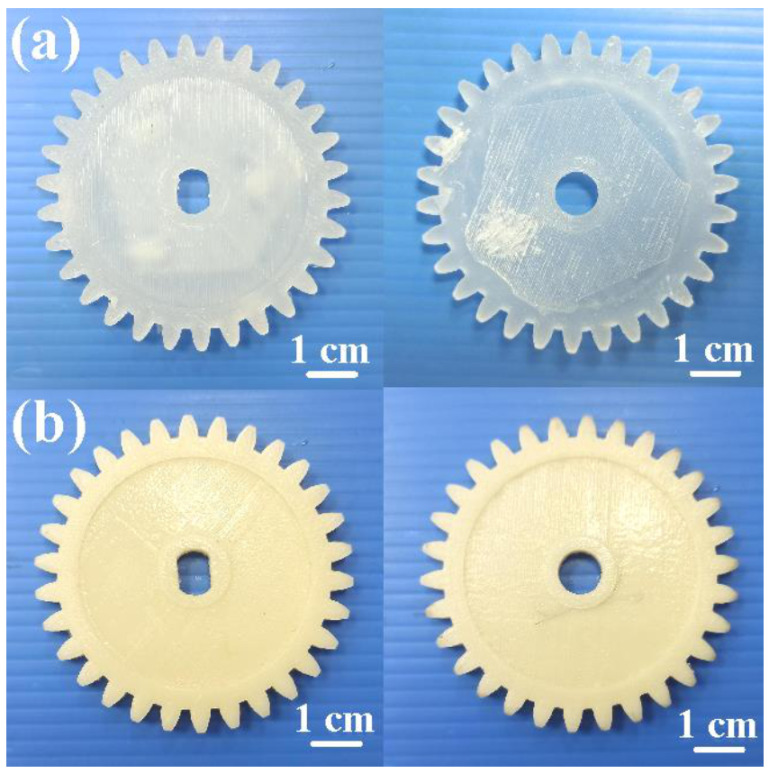
Spur gears fabricated by (**a**) epoxy and (**b**) polyurethane resins using DPVC technique. Driving gear (**left**) and passive gear (**right**).

Figure 16 shows typical spur gears printed with PLA, ABS, PC, and PA filaments. The distinct warpage of the printed gear was found due to uneven shrinkage [22]. It should be noted that two phenomena were found. One is that the deformation of the gear printed with the PC filament is the largest, followed by PA and ABS; the deformation angles are about 5.7 °, 2.2°, and 1.8°, respectively. Note that this drawback can be resolved by mounting an auxiliary heating plate on the printing head [23]. The other phenomenon observed is that the flatness of gears printed with PLA filament is better. Small batch production of prototypes via vacuum casting seems to be a good solution, since the cost of silicone rubber mold is at least ten times less than a conventional steel injection mold. In addition, the fatigue life of the polymer gear was greatly influenced by the lunker defects generated during the injection molding process. Note that no lunker defects were observed, which is widely observed with the polymer gears fabricated by plastic injection molding. Figure 17 shows the spur gears fabricated by epoxy and polyurethane resins using the DPVC technique. The results clearly show that the gears fabricated by DPVC have excellent flatness.

Polymer gears are usually designed with small tooth modules and operated in dry contact conditions for light loading transmissions [24]. Polymer gears involve three obvious failure types, including tooth root breakage, tooth wear, and tooth flank failure. In general, wear and thermal damages are widely observed in polymer gears in light loading conditions. To evaluate the wear resistance characteristics of gears fabricated by DPVC and AM technologies, an in-house abrasion testing machine was applied to investigate wear loss of the gear under 3000 rpm and an operating time of 120 min. The wear losses were discovered from the changes in the weight of gears before and after abrasion testing using a precision electronic scale.

**Figure 18:**
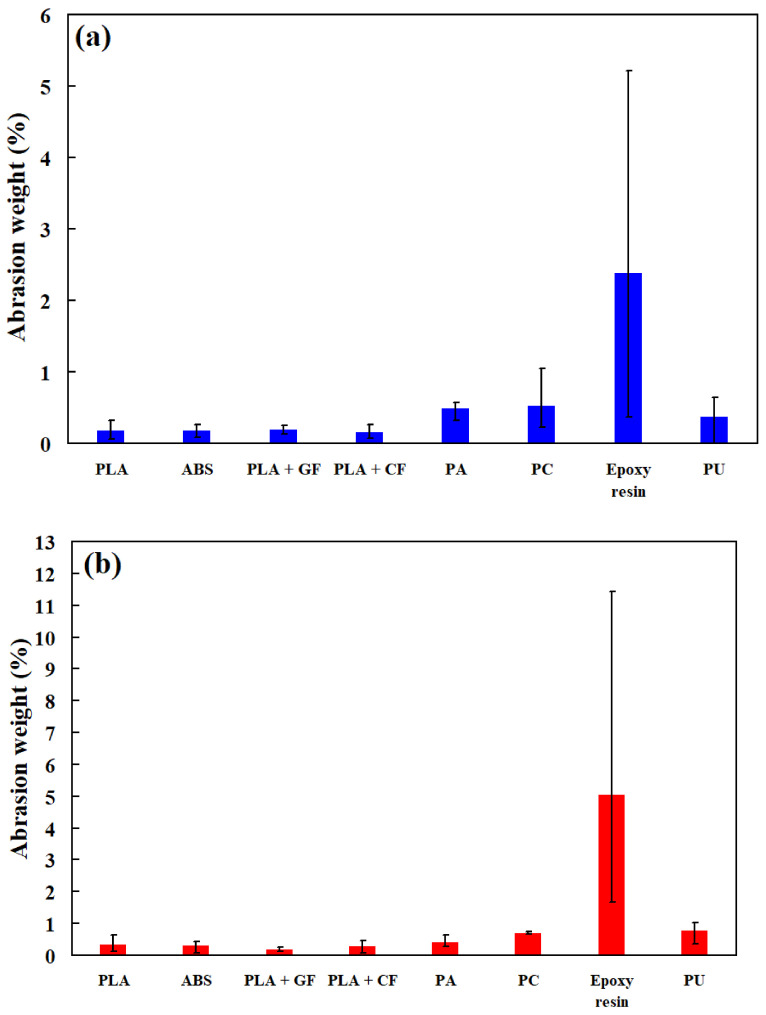
Abrasion weight percentage of gears fabricated with eight different materials (**a**) driving gear and (**b**) passive gear.

Figure 18 shows the abrasion weight percentage of gears fabricated with eight different materials for driving and passive gears. The average abrasion weight percentages of driving gears fabricated by filaments of PLA, ABS, 10 wt.% glass fiber-reinforced PLA, 10 wt.% carbon fiber-reinforced PLA, PA, PC, epoxy, and polyurethane resins are 0.173%, 0.182%, 0.192%, 0.155%, 0.485%, 0.524%, 2.379%, and 0.373%, respectively. In addition, the average abrasion weight percentages of passive gears fabricated by filaments of PLA, ABS, 10 wt.% glass fiber-reinforced PLA, 10 wt.% carbon fiber-reinforced PLA, PA, PC, epoxy, and polyurethane resins are 0.325%, 0.302%, 0.192%, 0.287%, 0.418%, 0.696%, 5.039%, and 0.761%, respectively.

**Figure 19:**
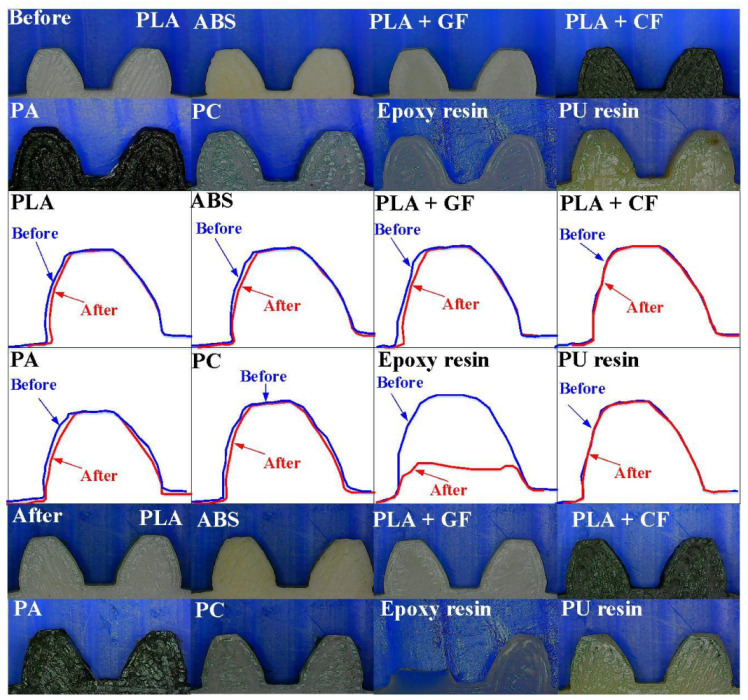
Corner wear evolution of gears fabricated with eight different materials.

Figure 19 shows the corner wear evolution of gears fabricated with eight different materials. It is evident that there is significant wear of the tooth surface. However, some common defects of gears (fisheye defects, debris frosting, pitting, or moderate pitting) were not found on the surface of the failed spur gears. 

**Figure 20:**
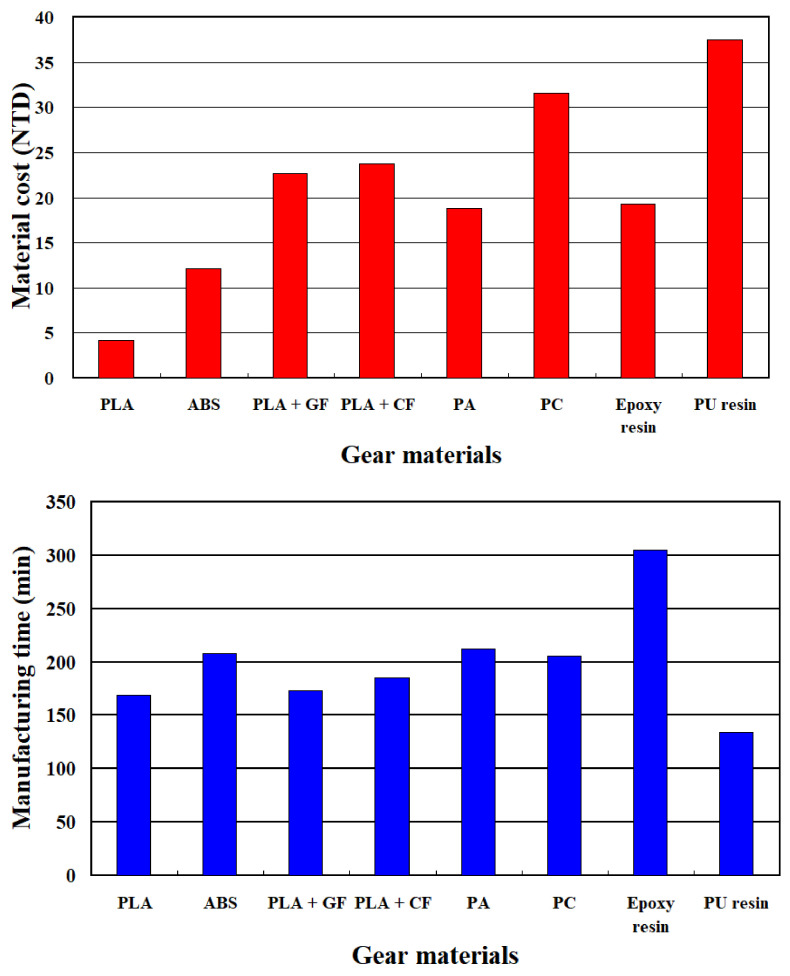
Materials cost and manufacturing time for gears fabricated with eight different materials.

Figure 20 shows the cost of materials and manufacturing time for gears fabricated with eight different materials. The results show that manufacturing times for gears fabricated with PLA, ABS, 10 wt.% glass fiber-reinforced PLA, 10 wt.% carbon fiber-reinforced PLA, PA, PC, epoxy resin, and polyurethane resin are 169, 208, 173, 185, 212, 206, 305, and 134 min, respectively. The costs of materials for gears fabricated with PLA, ABS, 10 wt.% glass fiber-reinforced PLA, 10 wt.% carbon fiber-reinforced PLA, PA, PC, epoxy resin, and polyurethane resin are 4.16, 12.13, 22.64, 23.75, 18.75, 31.62, 19.28, and 37.5 in new Taiwan dollars (NTD), respectively. 

Based on wear resistance, flatness, production time, and the materials cost of gears, four suggestions are proposed: (a) epoxy resin is not suitable for making gears since part of the teeth will be broken during abrasion test. The underlying reason for gear failure is that the material of polymer gears is fragile; (b) 10 wt.% glass fiber-reinforced PLA or 10 wt.% carbon fiber-reinforced PLA are recommended for making a small batch of gears for functional testing; (c) ABS, PA, or PC are not suitable for making gears because of the larger amount of deformation produced, and (d) polyurethane resin is also suitable to make gears for small quantity demand based on the inconspicuous deformation and abrasion weight percentage. In addition, the wear resistance of gears fabricated with polyurethane resin can be further enhanced by adding reinforcing fillers into base materials.

According to the aforementioned results, the findings of this study are very practical and provide potential applications in consumer electronics, automotive, aerospace engineering, medical, or architectural industries because this technique can be used to fabricate small batch production of polymer gears for functional testing at the research and development stage. The fabricated polymer gears can be further machined, such as by polishing, grinding, cutting, tapping, or drilling. In practice, pressure and temperature are the most significant variables in the differential pressure vacuum casting process. To achieve intelligent manufacturing during mass production of transmission components using VC technology, it is recommended that both pressure and temperature sensors are embedded in the cavity of the silicone rubber mold to monitor operational parameters during the differential pressure vacuum casting process. In this study, both epoxy resin and polyurethane resin were employed to manufacture polymer gears. Alternative polymers, such as polycarbonate, nylon, acrylonitrile butadiene styrene, or polypropylene were recommended for the manufacture of polymer gears. In addition, the mechanical properties of the fabricated polymer gears were dramatically affected by the intrinsic material properties of the molding material. Hence, the mechanical properties of the fabricated polymer gears can be further improved by adding reinforcing fillers, such as bentonite [25], silsesquioxanes, silica, alumina [26], zirconium dioxide, silicon dioxide [27], silicon carbide [28], silicon nitride [29], or molybdenum disulfide [30] into the matrix materials. These issues are currently being investigated and the results will be presented in a later study.

## 4. Conclusions

Polymer gears have been widely applied in transmission systems due to low noise and low costs compared to metal gears. The main purpose of this study was to characterize polymer gears fabricated by both DPVC and AM. The filling system of the silicone rubber mold was optimized by utilizing the numerical simulation software. Abrasion test equipment for evaluating spur gear life was designed and implemented. The main conclusions from the experimental work in this study are as follows:

1. The remarkable findings in this study are very practical and provide potential applications in the research and development stage because this technique can be used to fabricate small batch production of polymer gears for functional testing.
2. Notably, 10 wt.% glass fiber-reinforced PLA or 10 wt.% carbon fiber-reinforced PLA are suggested for the small batch production of gears for functional testing. ABS, PA, or PC are not suitable for making gears because they produce a larger amount of deformation.
3. Polyurethane resin is suitable for manufacturing polymer gears for small quantity demand based on the inconspicuous deformation and abrasion weight percentage. In addition, the wear resistance of gears fabricated with polyurethane resin can be further enhanced by adding reinforcing fillers into base materials.

## Figures and Tables

**Table 1:** Main numerical modeling parameters used in the numerical analysis.

Properties	Value
Filling time (s)	10
Material temperature (°C)	27
Mold temperature (°C)	27
Maximum injection pressure (kPa)	30

## Data Availability

The data presented in this study are available on request from the corresponding author.
